# New-Onset, Treatment-Resistant Inflammatory Bowel Disease after Administration of Secukinumab for Plaque Psoriasis: A Case Report and Review of the Existing Literature

**DOI:** 10.31138/mjr.030124.ntt

**Published:** 2024-03-31

**Authors:** Michail Krikelis, Evgenia Papathanasiou, George Leonidakis, Pavlos Pardalis, Spyridon Michopoulos, Evanthia Zampeli

**Affiliations:** 1 Greek Rheumatology Society and Professional Association of Rheumatologists, Private Practice, Greece,; 2 Gastroenterology Department, Alexandra General Hospital, Athens, Greece

**Keywords:** IL-17 inhibitor, inflammatory bowel disease

## Abstract

**Introduction:**

Aberrant activation of the IL-23/IL-17 axis leads to inflammatory phenotypes with overlapping clinical characteristics. Inhibition of IL-17 has mostly an anti-inflammatory effect, but sporadic cases of new-onset IBD have been reported.

**Case description:**

We present the case of a 65-year-old male patient with new-onset Crohn’s-like disease after treatment with secukinumab for skin psoriasis. Discontinuation of the IL-17 inhibitor and high-dose corticosteroid treatment were efficient initially, but a relapse was noted during corticosteroid tapering. Administration of certolizumab pegol did partially relieve the patient, but disease remission was only achieved with subcutaneous risankizumab therapy.

**Discussion:**

Clinical trials and real-world data indicate sporadic cases of new-onset IBD in patients receiving IL-17 inhibitors. Interestingly, our case is a “treatment-resistant” one since treatment with a biologic disease-modifying drug (bDMARD) usually leads to disease remission. As such, it is crucial to investigate the special characteristics of this clinical entity.

## INTRODUCTION

Psoriatic disease is a common diagnosis affecting 2–3% of the global population.^1^ Even though its exact pathophysiology remains to be clarified, it shares common molecular pathways with other immune-mediated diseases, such as axial spondylarthritis and inflammatory bowel disease (IBD). This common pathogenetic background is reflected in the overlap of clinical phenotypes among these entities. For example, in a recent meta-analysis, the odds ratio of psoriasis was 2.2 in patients with Crohn disease (CD) and 1.6 in patients with ulcerative colitis (UC), whereas the odds ratio of CD and UC in a population with psoriatic disease was 2.0 and 1.5, respectively.^2^ Whatever the clinical presentation, the aberrant activation of IL-23/IL-17 axis underlies the pathophysiology of these diseases and orchestrates the inflammatory response.^3^ In the psoriatic skin, superficial injuries damage keratinocytes and lead to antigen recognition by local dendritic cells. The activated dendritic cells either mature to plasmacytoid dendritic cells to produce type 1 interferons or migrate as myeloid dendritic cells to local lymphatic tissues (mostly areal lymph nodes), where they activate T-lymphocytes through the mediation of TNF-α, IL-17, IL-23, IL-22 and IL-1β. The Th1 and Th17 T-lymphocytes found in the psoriatic plaque are the result of direct differentiation of T-lymphocytes under the influence of the local pro-inflammatory environment or their migration from the local lymphatic tissues. In both cases, the final result is the overt production of IL-17 and the sustainment of a positive feedback loop characterised by the proliferation and activation of keratinocytes in the psoriatic plaque.^4^

A similar pathophysiological mechanism applies to the paradigm of IBD. Symbiotic/dysbiotic pathogens or auto-antigens exposed by mucosal micro-fissures are recognised by local dendritic cells and are presented to T-lymphocytes residing in the local lymphatic tissue (GALT) or in the areal lymph nodes. Differentiated Th17 T-lymphocytes invade the intestinal wall and promote inflammation through the excess production of IL-17. Another pathway includes the production of IL-23 by local macrophages, its direct action on local T-lymphocytes and their subsequent differentiation to a Th17 phenotype. IL-23 also interacts with γδ T-cells, innate lymphoid cells, neutrophils, and NK cells pointing to their role in intestinal inflammation.^5^

In both cases, the self-sustained production of IL-17, sometimes but not always stimulated by IL-23, leads to tissue damage through a high-affinity interaction of the molecule with its receptor. This far, six IL-17 types (A–F) which act in homodimers or heterodimers and bind to one of the five IL-17 receptors (R_A_-R_E_) have been recognised. Activation of the IL-17 receptors leads to the downstream production of IL-6 and IL-8, thus promoting the local aggregation of inflammatory cells.^3^ Thus, one would assume that inhibition of IL-17 could yield a considerable anti-inflammatory effect in diseases where the IL-23/IL-17 axis is disproportionally activated. Although this is the case for psoriatic skin lesions, evidence for IBD is contradictory. The neutralisation of IL-17A in murine models led to a phenotype with severe colitis.^6^ At the same time, clinical trials of IL-17 inhibitors reported sporadic cases of autoimmune intestinal inflammation.^7^ Last, randomised controlled trials (RCTs) of secukinumab in IBD patients led to exacerbation of patients’ symptoms and a higher incidence of fungal infections.^8^ The above led to the discovery that IL-17A physiologically sustains epithelial barrier function through the regulation of the tight junction protein occludin. IL-23R+ γδ T-cells are thought to be the main producers of this gut-protective IL-17A, even though its production seems to be partially dependent on IL-23.^9^

To contribute to the investigation of this Janus-faced cytokine, we discuss the case of a patient with plaque psoriasis and new-onset, treatment-resistant Crohn’s-like autoimmune colitis induced by treatment with the IL-17 inhibitor secukinumab. We also supplement our case with an up-to-date literature review about new-onset IBD in patients receiving IL-17 inhibitors.

## CASE DESCRIPTION

The patient is a 65-year-old male with psoriatic disease under treatment with the IL-17 inhibitor secukinumab. Before treatment, the patient suffered from extended, recent-onset plaque psoriasis (body surface area of 15%) and his disease spectrum did not include gastrointestinal, musculoskeletal, or other visceral symptoms (hidradenitis, uveitis, etc.). According to his medical history, the patient is a former smoker (20 pack-years), has a body mass index of 24 kg/m^2^ and is treated for heart failure (NYHA II, ejection fracture of 55%) due to ischemic heart disease (bypass surgery 8 years ago), abdominal aortic aneurysm (diameter of 4 cm with mural clot), dyslipidaemia, depressive disorder, and unilateral cataract. The patient is HLA-B27 negative, and his family history does not include IBD. He had already failed treatment with apremilast and, as per protocol, the treating dermatologist prescribed secukinumab at a dosage of 300 mg (induction with 300mg weekly for one month and maintenance with 300mg monthly).

**Figure 1.**
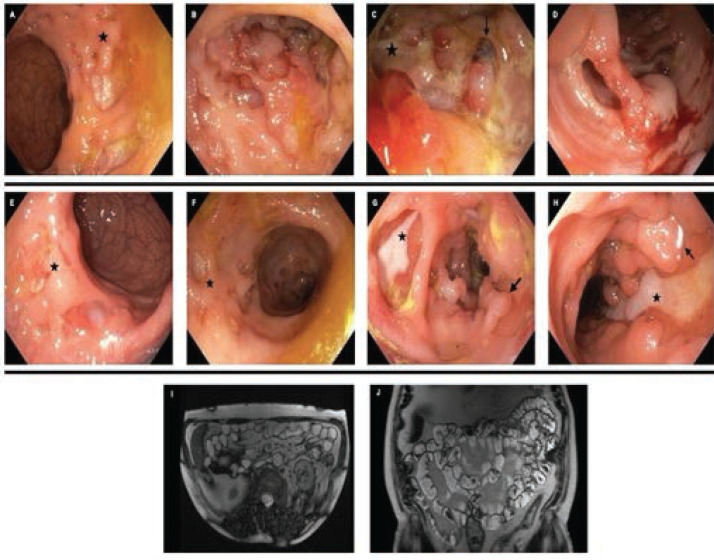
Endoscopic images at baseline **(A–D)** and after two months of corticosteroid therapy **(E–H)**. Magnetic resonance enterography images **(I–J)** under certolizumab pegol (CTZ) treatment. Initial sigmoidoscopy manifests widespread discontinuous inflammation with a patchy pattern (extent > 75%), mucosal oedema with exudates and an absent vascular pattern **(A, B)**, multiple large, deep ulcers with extended margins and focal necrosis **(B, C)**, secondary stenosis that cannot be passed **(C)** and pseudopolyps **(D)**. Despite high-dose corticosteroid treatment, the patient’s endoscopic findings reveal a decreased but persistent patchy inflammation (extent 10–30%) **(E, F)**, large linear and deep ulcers and pseudopolyps **(G, H)**. Under CTZ treatment, magnetic resonance enterography shows widespread transmural oedema in the colon marked as white areas in T1-weighted sequences **(I, J)**.

After completing the induction scheme, the patient reported abdominal pain and 5–6 daily episodes of bloody diarrhoea. He was admitted to the hospital and laboratory tests revealed leucocytosis, anaemia, thrombocytosis, moderately increased erythrocyte sedimentation rate (ESR) and C-reactive protein (CRP), while faecal calprotectin was disproportionately increased (**Table 1**). Further workup excluded infectious causes (including cytomegalovirus) and intestinal malignancy. Abdominal computed tomography (CT) showed bowel wall thickening from the descending colon to the caecum. Flexible sigmoidoscopy revealed numerous round, deep, invasive ulcers with elevated margins (**Figure 1A–D**). Endoscopy was strongly suggestive of severe Crohn’s disease, possibly triggered by secukinumab, which was subsequently discontinued. Due to both clinical and endoscopic severity, the patient was started on prednisolone (0.7 mg/kg/d) and his symptoms rapidly subsided.

**Table 1.** Patient’s clinical presentation, laboratory exams, and administered treatments at baseline and according to treatment schemes.

**Time frame**	**Baseline**	**1 month**	**4 months**	**7 months**
**Administered treatments**	none	Prednisolone 0.2 mg/kg/d	CTZ 200mg biw Prednisolone 5 mg/d	RIS 150mg / 3m
**Clinical appearance**	acute abdominal pain, 5–6 episodes of bloody diarrhoea per day	1–2 episodes of serous diarrhoea per day	1–2 episodes of serous diarrhoea per day	none
**Laboratory exams**	Hb 10.1 g/dl WBC 10,100 PLTs 437,000 ESR 52 mmHg CRP 25.3 g/dl (normal value: <5 g/dl) Calpro 5993 μg/g	Hb 9.7 g/dl WBC 8,400 PLTs 465,000 ESR 40 mmHg CRP 11.6 g/dl (normal value: <5 g/dl) Calpro 1052 μg/g	Hb 12.7 g/dl WBC 8,200 PLTs 364,000 ESR 38 mmHg CRP 10.5 g/dl (normal value: <5 g/dl) Calpro 765 μg/g	Hb 13.3 g/dl WBC 8,600 PLTs 270,000 ESR 18 mmHg CRP 5.3 g/dl (normal value: <5 g/dl) Calpro 152 μg/g
**Management**	High-dose prednisolone (0.7 mg/kg/d)	Initiation of CTZ 200 mg biw following initial schema	STOP CTZ; Initiation of RIS 150 mg / 3m following initial schema	Continuation of RIS in maintenance dose

Hb: haemoglobulin; WBC: white blood cell count; PLTs: platelet count; ESR: erythrocyte sedimentation rate; CRP: C-reactive protein (high sensitivity); Calpro: faecal calprotectin; CTZ: certolizumab pegol; biw: biweekly scheme; RIS: risankizumab.

A month later and whilst the patient was weaning off steroids, he presented with a new episode of abdominal pain and diarrhoea. Laboratory tests were still suggestive of systemic inflammation and faecal calprotectin was increased (**Table 1**). Endoscopy confirmed persistent inflammation with large, linear, deep ulcers accompanied by exudates, mucosal erosions and pseudopolyps (**Figure 1E–H**). Infectious causes were discarded anew. Steroid-dependency was considered, and treatment options discussed with the dermatologists. The patient was started on subcutaneous certolizumab pegol (induction with 400mg at 0-2-4 weeks and maintenance with 400mg eow), but minimal response was recorded at three months. This was also documented in the magnetic resonance enterography whilst on certolizumab pegol treatment (**Figure 1I–J**). Subsequently, certolizumab was discontinued and a month later, treatment with the anti-IL-23 antibody risankizumab was commenced (induction 150mg at 0 and 4 weeks and maintenance with 150mg every 12 weeks). Three months later, intestinal symptoms and psoriatic skin lesions were effectively controlled, whereas biochemical remission was also achieved (**Table 1**). Steroids were successfully suspended. After 12 months under risankizumab, the patient remains in remission, with a Harvey Bradshaw index (HBI) for intestinal inflammation equal to zero and a body surface index (BSI) for psoriasis equal to 1%.

## DISCUSSION

To our knowledge, this is the first case of a new-onset, treatment resistant inflammatory bowel disease, associated to IL-17 inhibitor, that was successfully treated with risankizumab. The patient did not respond to discontinuation of the IL-17 inhibitor. Steroids were offered and certolizumab pegol was commenced once steroid dependency was documented. However, certolizumab was ineffective, and remission was eventually achieved with the anti-IL-23 inhibitor risankizumab.

Such “treatment-resistant” cases are rare according to a recent review by Orzan et al.^4^ The most thorough review regarding new-onset IBD after treatment with IL-17 inhibitors, has been provided by Deng et al. In this retrospective study, 34 cases were included, most of which (91%) reported diarrhoea as the prevailing symptom. The patients were exposed to either secukinumab (79%) or ixekizumab (21%), whereas the most common indications were psoriasis (59%) and ankylosing spondylitis (21%). Endoscopic and histological findings were suggestive of ulcerative colitis or Crohn’s-like bowel inflammation in 46% and 36%, respectively. One third of patients had received anti-IL-17 treatment for 1–3 months before the onset of intestinal symptoms, thus pointing to an acute or sub-acute onset of the phenomenon. All reported cases discontinued the IL-17 inhibitor, but only 3% responded without treatment escalation. High-dose corticosteroids were administered in 32% of the cases, whereas a TNF-α inhibitor and an IL-23 inhibitor were added in 19% and 6.4% of cases, respectively. The rest received mesalamine (3.2%), corticosteroids plus mesalamine (13%), corticosteroids plus TNF-α inhibitor (19.4%) and corticosteroids plus ustekinumab (3.2%).^10^ The occurrence of IBD has been reported in clinical trials of IL-17 inhibitors with varying incidence. A recent meta-analysis by Truong et al. suggests a slightly increased incidence of new-onset IBD in RCTs with patients receiving IL-17 inhibitors.^11^ The calculated odds ratio (OR) of IBD in the treated population was two or three times higher than the placebo or the active comparator (mainly TNF-α inhibitor), respectively. However, it should be noted that in two thirds of the RCTs with IL-17 inhibitors, not a single IBD event was noted, while the overall observed frequency was calculated as low as 0.1–0.2/100 PY. Despite the rarity, the authors advocate that clinicians should be vigilant regarding the risk of IBD in clinical practice, since there is not a standard definition of an IBD-related adverse event, pre-existing IBD is reported in a few cases, trial populations are generally meticulously selected, and follow-up periods are usually short.^11^

Similar conclusions are drawn from research in real-world settings. In the medical database Northwestern Medicine Enterprise Data Warehouse (NMEDW), the frequency of secukinumab-induced, new-onset IBD, was 0.7%, whereas no cases were spotted in the ixekizumab-treated population. The Food and Drug Administration Adverse Event Reporting System (FAERS) also points out an increased frequency of new-onset IBD events in a secukinumab-treated population. The Australian Database of Adverse Event Notifications and the Canadian Vigilance Adverse Reaction Online Database report new-onset IBD cases in patients receiving secukinumab or ixekizumab, but no firm conclusions could be drawn due to low patient numbers.^12^

There is a low but not negligible rate of IBD occurrence when administering IL-17 inhibitors to axSpA patients and a high suspicion should be retained towards pre-existing or current gastrointestinal symptomatology in these patients. It is worth highlighting that clinically evident IBD has been observed in less than 15% of ankylosing spondylitis patients, whereas “silent” gut inflammation may accompany axSpA in up to 60% of cases.^13^ The “gut-joint axis hypothesis” integrates theories of dysbiosis and lymphocyte migration in order to interpret the pathophysiology underlying this co-existence.^14^ In light of the above, physicians who treat axSpA patients should give special emphasis on possible “red flags”, such as chronic persistent diarrhoea, abdominal pain recurrent oral aphthosis, constitutional symptoms or family history of IBD.^15^ When a high index of suspicion is present, some experts propose screening with a faecal calprotectin test before commencing an IL-17 inhibitor.^16^

In terms of management, discontinuation of the IL-17 inhibitor is of top priority. Thereafter, a case-by-case assessment is necessary if symptoms persist. Steroids usually ensure rapid remission. If intestinal inflammation is steroid-dependent or steroid-resistant, other treatment modalities should be considered. TNF-α inhibitors, IL-12/23 inhibitors and IL-23 inhibitors have shown efficacy, whereas JAK inhibitors may also be a promising treatment option in selected patients.^4,5^ However, when choosing the appropriate treatment for this condition, physicians should follow a treat-to-target strategy by simultaneously taking into consideration other manifestations of the pre-existing axSpA (uveitis, hidradenitis, etc).^17^

In conclusion, new-onset IBD is an infrequent but significant adverse event of IL-17 inhibition. Patients should be carefully screened for gastrointestinal inflammation before initiation of anti- IL-17 therapy. If IBD is suspected, IL-17 inhibitors should preferably be avoided. However, when such an adverse event does occur in clinical practice, the responsible IL-17 inhibitor should be discontinued and followed by a tailor-made corticosteroid course. In non-responsive or relapsing cases, suitable immunosuppression therapy should be offered.
